# Feasibility and clinical value of linear endoscopic ultrasonography imaging in the lower gastrointestinal subepithelial lesions

**DOI:** 10.1038/s41598-024-57130-x

**Published:** 2024-03-18

**Authors:** Li Tao, Yajun Chen, Qianqian Fang, Fan Xu, Qianwei Yu, Lijiu Zhang, Xiangpeng Hu

**Affiliations:** 1 Department of Gastroenterology, the Second Affiliated Hospital of Anhui Medical University, No. 678 Furong Road, Hefei, 230601 Anhui Province China; 2 Department of Gastroenterology, Fuyang Hospital of Anhui Medical University, Fuyang, 236000 Anhui Province China

**Keywords:** Gastrointestinal diseases, Intestinal diseases

## Abstract

Linear endoscopic ultrasonography (EUS) has been extensively utilized as a novel diagnostic and therapeutic modality across various fields. However, there have been relatively few studies focusing on lower gastrointestinal lesions. The aim of our study was to investigate the feasibility, safety and clinical value of linear EUS in the lower gastrointestinal subepithelial lesions. This was a retrospective study involving patients with lower gastrointestinal subepithelial lesions diagnosed by linear EUS from August 2019 to April 2023 at the Second Affiliated Hospital of Anhui Medical University. The data, including basic clinical information, linear EUS features, technical success rate, complications, and follow-up, were retrospectively collected and analyzed. A total of 69 patients with lower gastrointestinal subepithelial lesions underwent examination by linear EUS. Excluding the rectum, the technical success rate of linear EUS was 90.6% (29/32). Apart from the 7 patients whose diagnosis remained unknown, 3 patients with no abnormal EUS findings, and 3 patients failed the procedure, 56 patients were included in the final diagnostic performance analysis. The most common locations of the lesions were the rectum (37/56, 66.1%) and sigmoid colon (7/56, 12.5%). Based on endoscopy findings and pathological results, the most prevalent types of subepithelial lesions in the lower gastrointestinal tract were neuroendocrine tumor (NET) (12/56, 20.3%), lipoma (8/56, 13.6%) and extraluminal compression (8/56, 13.6%). The majority of lesions ranged in diameter from 1 to 3 cm (χ^2^ = 18.750, *p* < 0.001). After undergoing linear EUS examination, 36 patients received EUS-FNA (3/36), biopsy (5/36), endoscopic resection (25/36), or surgical excision (3/36) respectively. The pathological results of 29 patients were entirely consistent with the diagnosis made using linear EUS, with an 80.6% (29/36) diagnostic accuracy rate. Follow-up indicated that the lesions remained unchanged within 6–36 months. All patients tolerated the procedure well without any complications. In conclusion, linear EUS demonstrates technical feasibility, safety, and a high diagnostic accuracy for subepithelial lesions in the lower gastrointestinal tract.

The subepithelial lesions of the gastrointestinal tract are becoming increasingly widespread in the clinic and most cases are incidentally discovered during endoscopic examination^1^. Typically, these lesions may originate not only from the gastrointestinal tract walls but also from the compression of extrinsic structures. Intramural lesions can arise across any layer of the gastrointestinal wall, ranging from deep mucosa to serosa^2^. Certain mucosal lesions, such as polyps and cancers, originate from deeper layers of the mucosa, resembling submucosal lesions, thereby posing challenges for identification. The widespread use of colonoscopy has led to arise in the detection of subepithelial lesions in the lower gastrointestinal tract^3,4^. As the type of treatment and prognosis vary depending on the type of lesion, achieving an accurate diagnosis is of paramount importance.

Endoscopic ultrasonography (EUS) is a combination of endoscopy and intraluminal ultrasonography^5^. This technique enables visualization of the gastrointestinal tract lining through the endoscope, while ultrasound allows for the visualization of the gastrointestinal tract walls, surrounding organs, and blood vessels^6,7^. By utilizing EUS, it becomes possible to differentially diagnose conditions by documenting the originating gastrointestinal wall layers and their associated echogenic characteristics^8^.

As access to EUS becomes more widespread, it is emerging as a promising diagnostic modality for evaluating gastrointestinal lesions. Presently, reports on the use of linear EUS in lower gastrointestinal subepithelial lesions are primarily limited to case reports and small case series^9,10^. A recent study has demonstrated that many rectal and anal diseases, including perianal abscesses, fistulae, polyps, and neoplastic lesions, can be well-visualized and evaluated using linear EUS^11^. Using linear EUS, cytological/histological confirmation can be obtained through EUS-guided fine needle aspiration/biopsy (EUS-FNA/FNB)^8^. Results of a small-sample study showed EUS-FNB was 92% accurate in predicting the diagnosis compared to FNA, which achieved a correct diagnosis only in 58% of rectosigmoid lesions^12^. However, due to the challenges associated with the oblique endoscope view of linear EUS, its application in the lower gastrointestinal tract, aside from the rectum, has been rarely reported. Hence, this study aims to investigate the feasibility and safety of linear EUS in the lower gastrointestinal tract. Importantly, the diagnostic accuracy of linear EUS in lower gastrointestinal subepithelial lesion were evaluated. Our study shows that linear EUS exhibits high diagnostic accuracy for subepithelial lesions in the lower gastrointestinal tract.

## Methods

### Patients

This was a retrospective study. From August 2019 to April 2023, a total of 69 patients with lower gastrointestinal subepithelial lesions who underwent linear EUS were collected from the Endoscopy Center of the Second Affiliated Hospital of Anhui Medical University. Before undergoing linear EUS, all patients had previously undergone colonoscopy. Excluding 7 patients with unknown diagnosis (who refuse further examination), 3 patients with no abnormal EUS findings, and 3 patients failed the procedure, 56 patients were included in the final diagnostic performance analysis.

### Equipment and examination

Linear EUS (EU-ME2, GF-UCT260, Olympus, Tokyo, Japan; EG-3270UTK, Pentax, Tokyo, Japan) was utilized for the examination. The probe frequency is dynamically adjusted depending on the distance of the lesion from the probe and whether there is extraluminal compression, most of which is 7.5 MHz, occasionally 5 MHz or 9 MHz. All patients signed informed consent for EUS examination. To prepare for linear EUS, 654–2 (10 mg) or diazepam (10 mg) was administered as premedication 30 min before the procedure. Given that the accuracy of EUS diagnosis heavily relies on the experience of the endoscopists, all patients in this study underwent examination and diagnosis by two experienced endoscopists (each with experience of > 1000 EUS examinations) to minimize bias. Clinical and colonoscopic information was given before EUS evaluation. However, endoscopists were blinded to the pathological results of these lesions. The procedure was conducted through an endoscope inserted into the anus. A downward angle allowed for viewing the rectal lumen, followed by insufflation with air and advancing the endoscope. The transition from the rectum to the recto-sigmoid junction was relatively straightforward. However, upon reaching the sigmoid colon, splenic flexure, and hepatic flexure, the direction of endoscopic advancement may not have been visible due to relatively large angles, which could be mitigated by adjusting body position and applying abdominal pressure. It was crucial to navigate these areas slowly and gently. Rotating the endoscope axis while entering the mirror was essential. In the descending colon, transverse colon, and ascending colon, visualizing the intestinal lumen before proceeding forward was necessary. Once the lesion was reached and the gas was evacuated, the water pump was employed to inject saline into the intestinal cavity, completely immersing the lesion. Throughout the process, it was important to bear in mind the distinction between the oblique endoscope view and direct vision.

The data, encompassing basic clinical information, linear EUS features, technical success rate (the success rate of operating linear EUS to reach the lesion location), were retrospectively collected and analyzed. The diagnosis and management were determined according to guidelines of the American Society for Gastrointestinal Endoscopy (ASGE)^3^. Subsequent treatments were chosen based on linear EUS imaging diagnosis of lesion, including options such as follow-up, biopsy, EUS-FNA, endoscopic resection, or surgical excision. Biopsy and postoperative pathological results were compared with the diagnoses obtained via linear EUS imaging. Patients not undergoing immediate intervention were periodically followed up. The primary outcome of this study was the diagnostic accuracy of linear EUS imaging in lower gastrointestinal subepithelial lesions. The secondary outcome included the assessment of technical success rate, safety and feasibility.

### Statistical analysis

The data processing was performed using the SPSS 22.0 statistical software. Categorical data are displayed as number (n) or percentage (%) and were compared using the *Chi-Square Test*. Quantitative data are expressed as the mean ± standard deviation (SD) and were compared using the *t*-test. A *p* value less than 0.05 was considered statistically significant.

### Ethical approval and consent to participate

This study was reviewed and approved by the Institutional Review Board of the Second Affiliated Hospital of Anhui Medical University (SL-YX2023-181), and conforms to the principles stated in the Declaration of Helsinki.

## Results

### Clinical features

A total of 69 patients with lower gastrointestinal subepithelial lesions underwent examination by linear EUS, resulting in 3 procedure failures. Exception the rectum, the technical success rate of linear EUS in reaching the lesion location was 90.6% (29/32). The final diagnostic performance analysis comprised 56 patients, including 29 males and 27 females, with an average age of 53.0 ± 12.04 years (range: 22–79 years). The average diameter of all lesions measured 15.95 ± 9.82 mm (range: 4.6–45.1 mm). The most frequent locations of the lesions were the rectum (37/56, 66.1%) and sigmoid colon (7/56, 12.5%), followed by transverse colon (4/56, 7.1%) and ascending colon (4/56, 7.1%) (Table 1).

**Table 1:** Distributions of subepithelial lesions in the lower gastrointestinal tract.

Lesions	Cecum	Ascending colon	Hepatic flexure	Transverse colon	Descending colon	Sigmoid colon	Rectum	Total	Percentage
NET	–	–	–	–	–	–	12	12	20.3
Lipoma	1	4	–	1	–	1	1	8	13.6
Extraluminal compression	–	–	1	–	1	1	5	8	13.6
Cancer	–	–	–	–	–	1	6	7	11.9
Polyps	–	–	–	–	–	2	2	4	6.7
Inflammation	–	–	–	–	–	–	4	4	6.7
Cyst	–	–	–	3	–	–	–	3	5.1
Varix	–	–	–	–	–	–	2	2	3.4
Glomus	–	–	–	–	–	2	–	2	3.4
PCI	–	–	1	–	–	–	1	2	3.4
GIST	–	–	–	–	–	–	2	2	3.4
Schwannoma	–	–	–	–	–	–	1	1	1.7
Endometriosis	–	–	–	–	–	–	1	1	1.7
Total	1	4	2	4	1	7	37	56	–
Percentage	1.8	7.1	3.6	7.1	1.8	12.5	66.1	–	100

**Figure 1:**
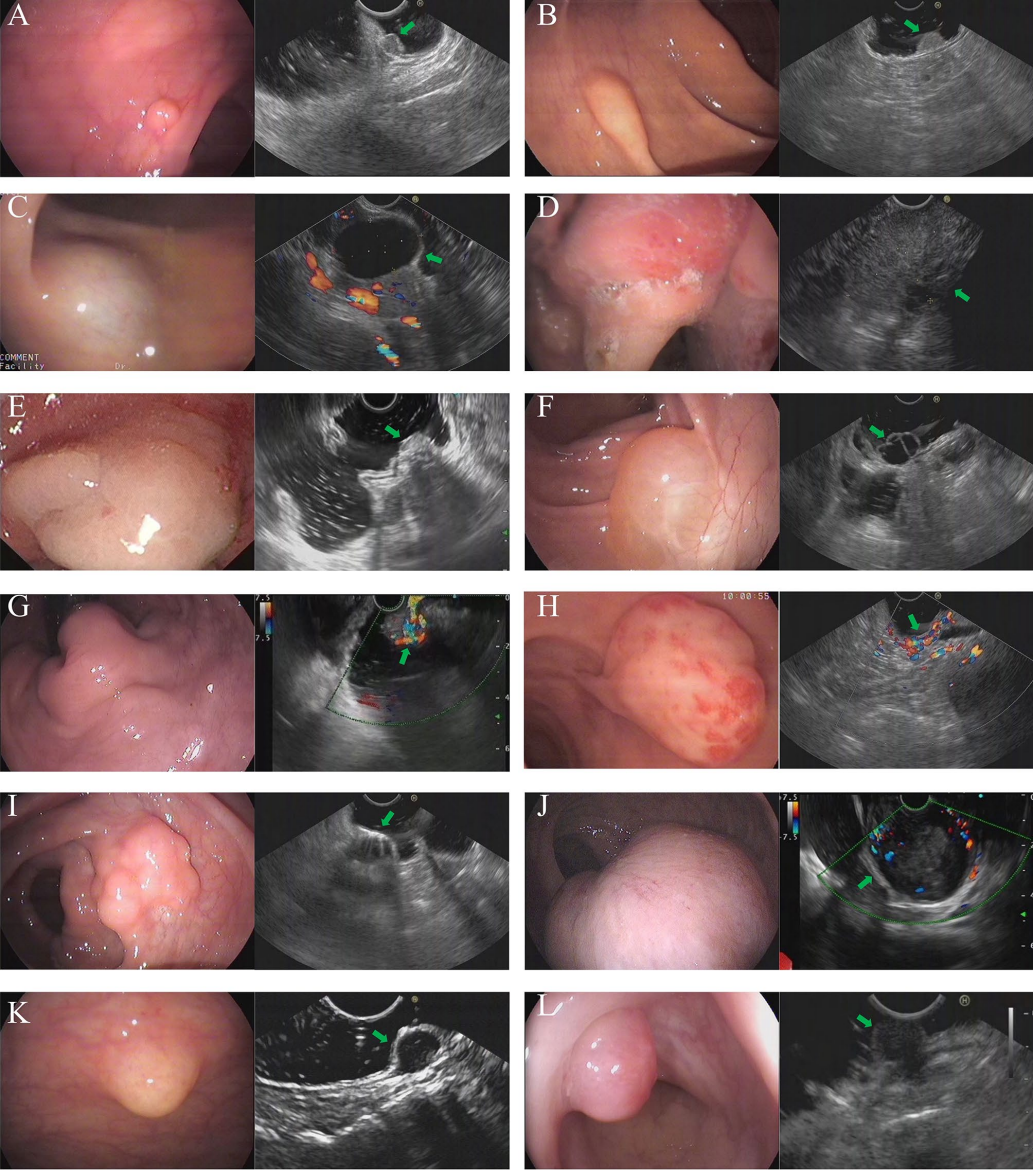
Endoscopic and ultrasonographic images of lower gastrointestinal subepithelial lesions. (**A**) Neuroendocrine tumor (NET); (**B**) Lipoma; (**C**) Renal cyst extraluminal compression; (**D**) Cancer; (**E**) Inflammation; (**F**): Cyst; (**G**) Varix; (**H**): Glomus; (**I**) Pneumatosis cystoides intestinalis (PCI); (**J**) Gastrointestinal stromal tumor (GIST); (**K**) Schwannoma; (**L**) Endometriosis.

Based on the findings from endoscopy and pathological results, the most prevalent types of lower gastrointestinal subepithelial lesions were neuroendocrine tumor (NET) (12/56, 20.3%), all of which were situated in the rectum. Subsequently, other common types included lipoma (8/56, 13.6%), extraluminal compression (8/56, 13.6%), and cancer (7/56, 11.9%). Additionally, various other observed lesions in the study comprised polyps, inflammation, cysts, varices, glomus, among others. These outcomes are visually displayed in Fig. 1 and Table 1.

### Linear EUS features

Linear EUS revealed extraluminal compression in 8 patients, attributed to external pressure organs include renal cysts, uterus and prostate. Among the remaining 48 patients, the linear EUS features varied. Most lesions ranged in diameter from 1 to 3 cm (*χ*^2^ = 18.750, *p* < 0.001) (Table 2). Out of 12 cases of NET, 9 were less than 1 cm in diameter (*χ*^2^ = 15.750, *p* < 0.001). Furthermore, lipomas were concentrated in the 1 to 3 cm in diameter range (*χ*^2^ = 10.500, *p* = 0.005). As depicted in Table 3, the most common sites of origin of NET were the muscularis mucosa and submucosal with a distinct border (*χ*^2^ = 16.889, *p* = 0.001). Nearly all lipomas originated from the submucosa with a distinct border (*χ*^2^ = 22.667, *p* < 0.001). Additionally, all cancer and polyps originated from the mucosa, with disrupted mucosal continuity. Cysts, varices, pneumatosis cystoides intestinalis (PCI) and schwannoma originated from the submucosa, while glomus, gastrointestinal stromal tumor (GIST), and endometriosis originated from the muscularis propria. In mucosal lesions, polyps and inflammation appeared hyperechoic while cancers were hypoechoic. The majority of NETs were visualized as hypoechoic, with 2 cases being hyperechoic (*χ*^2^ = 30.222, *p* < 0.001). All lipomas were hyperechoic (*χ*^2^ = 32.000, *p* < 0.001). Conversely, all cysts were anechoic. The comprehensive results can be found in Table 4.

**Table 2:** Sizes of subepithelial lesions in the lower gastrointestinal tract.

Lesions	< 1 cm	1–3 cm	> 3 cm	Total	*P*	*X*^2^
NET	9	3	–	12	< 0.001	15.750
Lipoma	2	6	–	8	0.005	10.500
Cancer	–	4	3	7	0.062	5.571
Polyps	1	2	1	4	0.687	0.750
Inflammation	3	1	–	4	0.072	5.250
Cyst	–	3	–	3	/	/
Varix	–	2	–	2	/	/
Glomus	–	2	–	2	/	/
PCI	–	2	–	2	/	/
GIST	–	–	2	2	/	/
Schwannoma	1	–	–	1	/	/
Endometriosis	–	1	–	1	/	/
Total	16	26	6	48	< 0.001	18.750

**Table 3:** Originating layers of the lesions on endoscopic ultrasonography (EUS).

Lesions	Mucosa layer	Muscularis mucosa layer	Submucosal layer	Muscularis propria layer	Total	*P*	*X*^2^
NET	–	5	7	–	12	0.001	16.889
Lipoma	–	1	7	–	8	< 0.001	22.667
Cancer	7	–	–	–	7	< 0.001	28.000
Polyps	4	–	–	–	4	/	/
Inflammation	2	–	2	–	4	/	/
Cyst	–	–	3	–	3	/	/
Varix	–	–	2	–	2	/	/
Glomus	–	–	–	2	2	/	/
PCI	–	–	2	–	2	/	/
GIST	–	–	–	2	2	/	/
Schwannoma	–	–	1	–	1	/	/
Endometriosis	–	–	–	1	1	/	/
Total	13	6	24	5	48	< 0.001	25.556

**Table 4:** Echogenicity of the lesions on endoscopic ultrasonography (EUS).

Lesions	Hyperecho	Isoecho	Hypoecho	Anecho	Total	*P*	*X*^2^
NET	2	–	10	–	12	< 0.001	30.222
Lipoma	8	–	–	–	8	< 0.001	32.000
Cancer	–	1	6	–	7	< 0.001	18.857
Polyps	4	–	–	–	4	/	/
Inflammation	3	1	–	–	4	/	/
Cyst	–	–	–	3	3	/	/
Varix	1	–	–	1	2	/	/
Glomus	–	1	1	–	2	/	/
PCI	–	–	2	–	2	/	/
GIST	–	–	2	–	2	/	/
Schwannoma	–	–	1	–	1	/	/
Endometriosis	–	–	1	–	1	/	/
Total	18	3	23	4	48	< 0.001	33.556

### Diagnostic accuracy of linear EUS

Out of these, 36 patients underwent histological confirmation by EUS-FNA (3/36), biopsy (5/36), endoscopic resection (25/36), or surgical excision (3/36) pathology. The diagnostic accuracies of linear EUS for lower gastrointestinal lesions are presented in Table 5. Postoperative pathological results and biopsies from 29 patients (29/36) were entirely consistent with the initial linear EUS diagnosis, demonstrating an 80.6% diagnostic accuracy. Linear EUS exhibited a 100% diagnostic accuracy for lipoma (1/1), cyst (2/2), glomus (1/1), and GIST (2/2), and achieved a 91.7% accuracy for NET (11/12). Additionally, the diagnostic accuracy of linear EUS was 85.7, 75 and 75% for cancer (6/7), polyps (3/4) and inflammation (3/4), respectively. There were 7 cases of misdiagnosis, which included 1 case each of inflammation, PCI and schwannoma misdiagnosed as NET, 1 case of NET and polyps misdiagnosed as lipoma, 1 case of cancer misdiagnosed as polyps, and 1 case of endometriosis misdiagnosed as GIST.

**Table 5:** Diagnostic rates of endoscopic ultrasonography (EUS) diagnosis based on final histopathology of the lesions.

Pathological results	Number of pathological diagnoses	Number of EUS diagnoses	Number of consistent diagnoses between pathology and EUS	Diagnostic consistency rate (%)
NET	12	14	11	91.7
Lipoma	1	3	1	100
Cancer	7	6	6	85.7
Polyps	4	4	3	75
Inflammation	4	3	3	75
Cyst	2	2	2	100
Glomus	1	1	1	100
PCI	1	–	–	0
GIST	2	3	2	100
Schwannoma	1	–	–	0
Endometriosis	1	–	–	0
Total	36	36	29	80.6

### Follow-up

Among these were 20 cases involving submucosal lesions that did not undergo endoscopic therapy or surgery, excluding cases with extraluminal compression. Out of these, 12 cases were monitored through endoscope or EUS for a span of 6–36 months. The findings revealed that the lesions reminded unchanged during the follow-up period. Notably, no complications associated with lower gastrointestinal linear EUS examinations were reported among any of the patients.

## Discussion

With the advancement of colonoscope and imaging examination, the reported incidence of subepithelial lesions in the lower gastrointestinal tract has increased^4,13^. In instances where lower gastrointestinal subepithelial lesions are encountered during colonoscopy and prove difficult to identify, EUS can be considered an ideal method for further examination as it furnishes valuable information concerning the location of the lesions (intramural or extramural), size, and echogenic characteristics^14^.

There are three EUS methods for detecting lower gastrointestinal subepithelial lesions: miniprobe EUS, radial EUS and linear EUS^15^. Compared with the other two types, the great advantage of linear EUS is that lesions and deeper layers can be better visualized on the same image. An additional advantage of linear EUS is the ability to sample the tissue by EUS-FNA/FNB^8^. Currently, most studies have concentrated on mini-probe EUS or radial EUS^4,11–13,16^, with fewer investigations employing linear EUS to explore lower gastrointestinal lesions. In a recent study, linear EUS was solely utilized in the rectum^11^. However, there are minimal relevant studies on the application of linear EUS for the entire lower gastrointestinal tract due to equipment requirement and operational challenges. In our study, linear EUS was employed to investigate lower gastrointestinal subepithelial lesions. The findings revealed that out of 69 patients who underwent linear EUS, 3 experienced procedural failures. Challenges were encountered in the hepatic region (1 patient) and sigmoid colon (2 patients), respectively. Except for the rectum, the operational success rate of linear EUS stood at 90.6% (29/32). This high technical success rate suggests that linear EUS is a viable method for examining the lower gastrointestinal tract, with the exception of the rectum.

The study demonstrates that linear EUS is capable of clearly displaying the adjacent structure of the infrasonic and pelvic compartments. Imaging from the upper rectum allows visualization of the left infrasonic compartment, retroperitoneal structures, and bowel loops^17^. However, there are numerous subepithelial lesions in the lower gastrointestinal tract above the rectum, which also require identification using linear EUS. When a subepithelial lesion is detected, accurate determination of its etiology is crucial as each type of subepithelial lesion requires different treatments, follow up, and prognosis. If extraluminal compression arises from a normal adjacent structure or organ, no further examination is necessary. In our study, linear EUS revealed extraluminal compression in 8 patients, attributed to external pressure organs such as renal cysts, uterus, and prostate, for which no further treatment was required. Meanwhile, 3 patients exhibited no abnormal findings under linear EUS. On one hand, it is possible that the 3 lesions may have been missed due to the existence of blind areas in the oblique endoscope view. On the other hand, the potential for extraluminal compression could not be excluded.

Based on the features observed with linear EUS, it is possible to diagnose cysts or lipomas without further tests or biopsies. Follow-up may only be necessary for selected patients, without invasive or aggressive intervention. However, when a hypoechoic subepithelial lesion is visible in the submucosal or muscularis propria, the differential diagnosis may include NET, GIST, leiomyoma, endometriosis, or schwannoma. Prior studies have shown that the diagnostic accuracy for lesions in the submucosal or muscularis propria layer using EUS alone is only around 50%^18^. In our study, pathological diagnoses were compared with the results obtained using linear EUS. The pathological outcomes of 29 patients were entirely consistent with the linear EUS diagnosis, resulting in an 80.6% (29/36) diagnostic accuracy. Rare lesions such as glomus, schwannoma, and endometriosis lacked distinctive features in linear EUS and appeared similar to common submucosal lesions, leading to potential misdiagnoses under linear EUS. Furthermore, the diagnostic accuracy of linear EUS is closely linked to operator skill, equipment, and clinical experience^19^. Thus, the diagnosis of subepithelial lesions must be combined with the patient’s medical history and relevant tests, especially pathological examinations^20^. Consequently, linear EUS-guided tissue acquisition may play a crucial role in distinguishing between these subepithelial lesions.

The oblique endoscope view of linear EUS can lead to increased entry difficulty and a heightened risk of perforation. In the lower gastrointestinal tract, negotiating the excessively long and freely mobile sigmoid colon is challenging, and the acute angles at the hepatic flexure and splenic flexure further exacerbate passage difficulties. During the operation, operators must delicately manipulate and control the lens while avoiding large movements, abrupt rotations, and forceful entries, especially through the aforementioned areas. The most critical aspect is to refrain from blindly conducting forced operations. Intravenous anesthesia or sedative drugs are recommended for patients undergoing linear EUS. In our study, all patients received antispasmodic or sedative treatment before the examination, and no significant discomfort was experienced during the procedure. Notably, no complications were reported among any of the patients who underwent lower gastrointestinal linear EUS examinations. Taken together, these results provided evidence that linear EUS is technically feasible, safe, and exhibits high diagnostic accuracy for subepithelial lesions in the lower gastrointestinal tract.

This study presents several limitations. Firstly, it was a retrospective study, potentially introducing selection bias during data collection. Secondly, our study was confined to a single-center. Thirdly, the small sample size further limited the scope of the study. Future analyses would benefit from larger prospective, multicenter studies to provide additional insights and comprehensive analysis of the findings.

## Conclusion

In summary, this retrospective study investigated the effect of linear EUS imaging in lower gastrointestinal subepithelial lesion. Our results demonstrated linear EUS exhibits high diagnostic accuracy for subepithelial lesions in the lower gastrointestinal tract. The evidence suggests that linear EUS is technically feasible and safe in the lower gastrointestinal tract. It can effectively aid in the differentiation of such lesions. Given its significant clinical application value, promotion of the use of linear EUS is warranted.

## Data Availability

The datasets used and/or analysed during the current study available from the corresponding author on reasonable request.
